# Supplementary material for: AFM Images of Viroid-Sized Rings That Self-Assemble from Mononucleotides through Wet–Dry Cycling: Implications for the Origin of Life
Source: Life (Basel). 2020 Nov 30;10(12):321. doi: 10.3390/life10120321 (PMC7760185; doi:10.3390/life10120321)
Supplement: Supplementary file 1 [file life-10-00321-s001.pdf]

life

MDPI

Supplementary Materials:

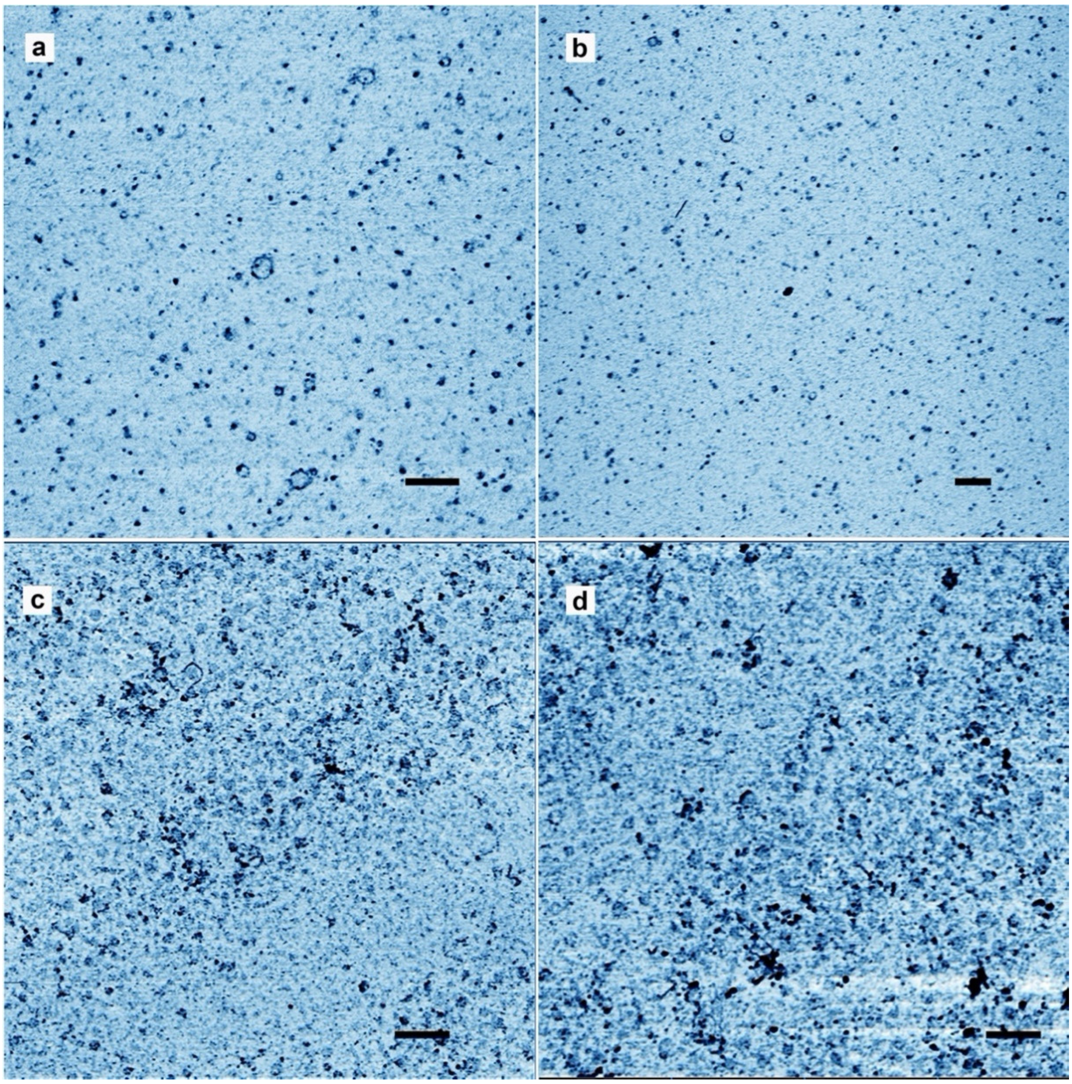

Figure 1. Additional data for AU mixtures. Rings were abundant after wet-dry cycling. The four micrographs show a mica surface which was exposed to a solution of AMP and UMP that had been cycled three times on a glass substrate. The images shown were from two different experiments in two different laboratories. Both display rings, but the amount of material deposited was different, making c) and d) not as clear as a) and b). The scale bars shows 200 nm.

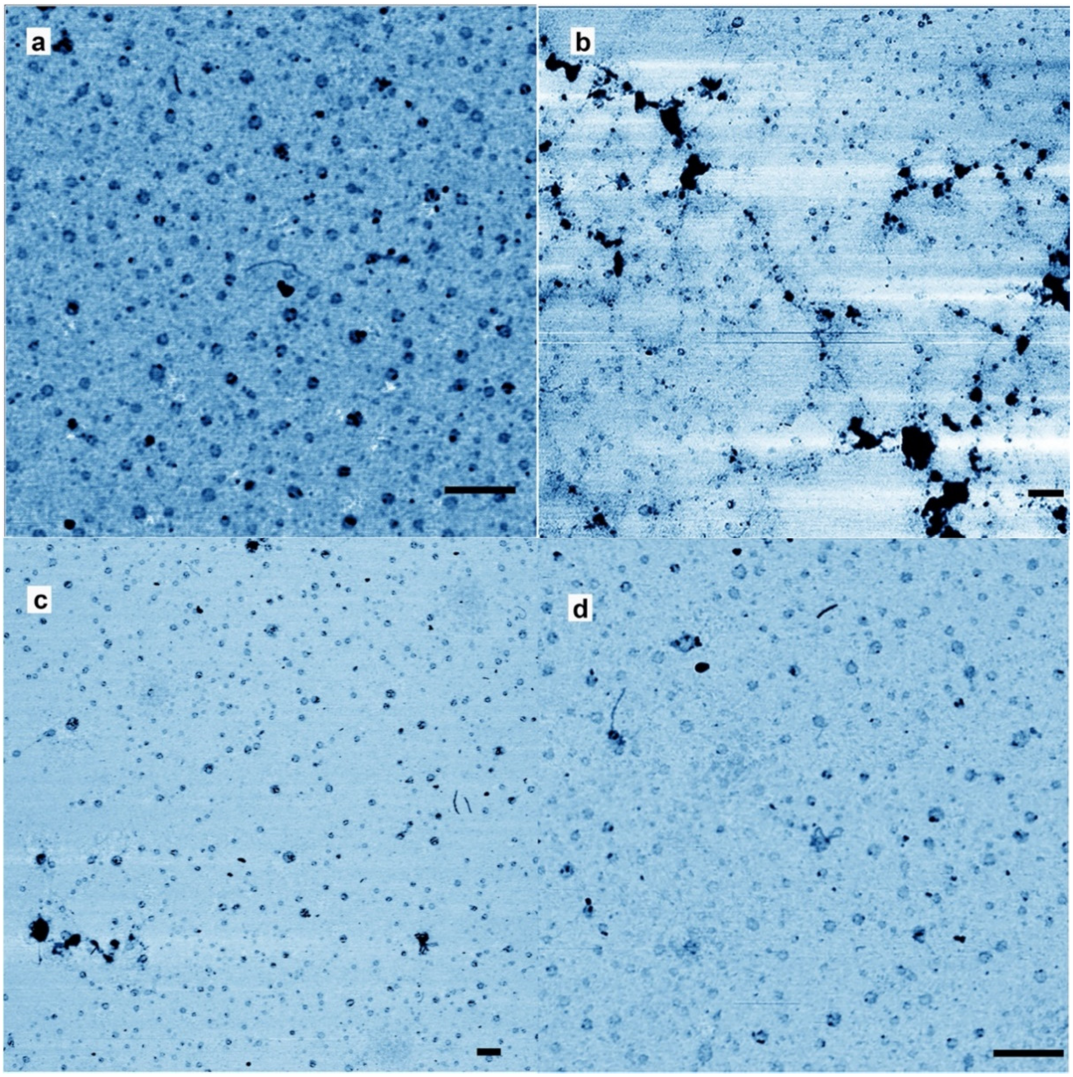

Figure 2. Additional data for GC mixtures. Rings were abundant after wet-dry cycling. The four micrographs show a mica surface on which a mixed solution of GMP and CMP had been cycled three times. The scale bars show 200 nm.

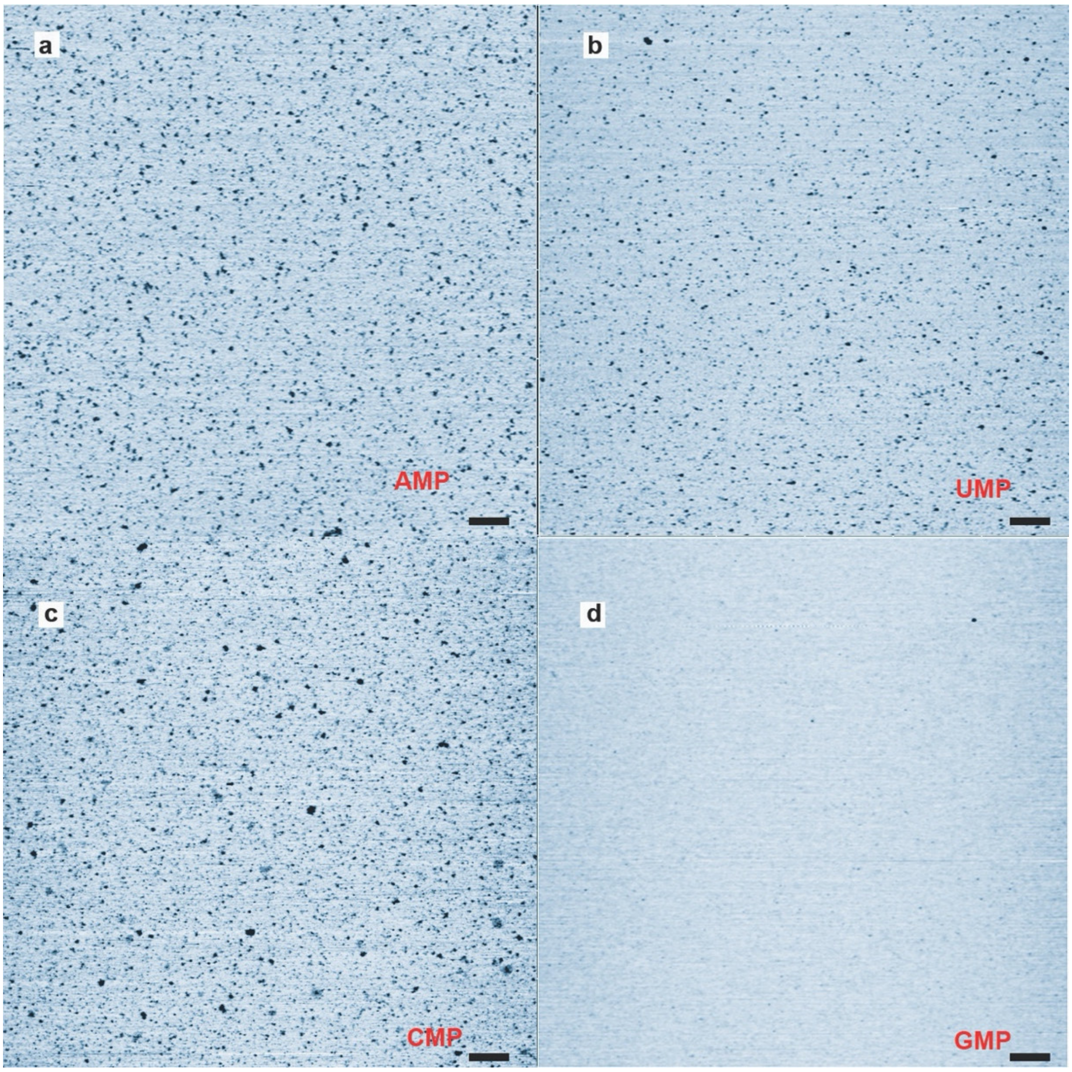

Figure 3. Representative AFM images of controls with deposits from uncycled 10 mM mononucleotides solutions on mica with a) AMP, b) UMP, c) CMP and d) GMP. The protocol was the same as that used for in Fig. S1, but without any wet-dry cycling. A few microliters of solution were added to a freshly cleaved mica surface, left there for 30 seconds and then rinsed with pure water. Only particles were apparent, showing that the solutions did not contain rings at the outset. We sampled at least 10 different spots across each sample. The scale bars show 200 nm.

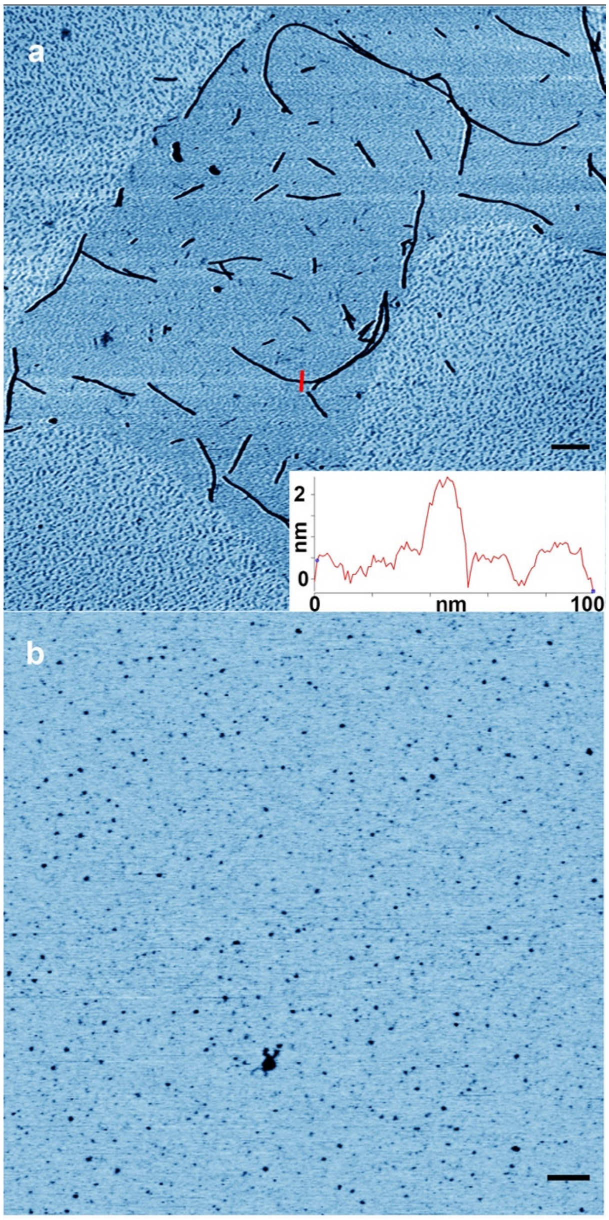

Figure 4. Products from room temperature wet-dry cycling of a) 1:1 GC and b) 1:1 AU mixture solutions. The cross section in a) shows that the GC fibrils are more than 2 nm thick and 14 nm wide, significantly different from the rings observed with wet-dry cycling at 80 °C. The fragmented forms and the mostly linear shapes suggests that the structures are soft crystals rather than polymers. No ring structures were observed with the GC or AU mixtures. The scale bars shows 200 nm.
